# Effect of low dose ω-3 poly unsaturated fatty acids on cognitive status among older people: a double-blind randomized placebo-controlled study

**DOI:** 10.1186/2251-6581-13-34

**Published:** 2014-02-07

**Authors:** Mohammad Jafar Mahmoudi, Mona Hedayat, Farshad Sharifi, Mojde Mirarefin, Neda Nazari, Neda Mehrdad, Mayam Ghaderpanahi, Yaser Tajalizadekhoob, Zohre Badamchizade, Bagher Larijani, Sudabeh Alatab, Mahtab Alizadeh, Seyed Masood Arzaghi, Baharak Najafi, Hossein Fakhrzadeh

**Affiliations:** 1 Tehran University of Medical Sciences, Tehran, Iran; 2 Elderly Health Research Center, Endocrinology and Metabolism Population Sciences Institute, Tehran University of Medical Sciences, No 4, Ostad Nejatollahi Street, Engelab Avenue, Tehran, Iran; 3 Endocrinology and Metabolism Research Center, Tehran, Iran; 4 Kahrizak Charity Foundation, Tehran, Iran

## Abstract

**Background:**

Cognitive impairment is a prevalent health problem in older people and its global prevalence tends to increase parallel to the extended life expectancy in world. The beneficial effect of ω-3 PUFAs on cognitive impairment has been demonstrated in some experimental and cohort studies. In this study we aimed to assess the effect of low dose docosahexaenoic acid (DHA) and eicosapentaenoic acid (EPA) supplementation on cognitive status in the elderly.

**Methods:**

In a double-blind, randomized placebo-controlled study, 199 individuals aged ≥65 years with normal or mild to moderate cognition impairment were assigned to receive either 180 mg of DHA plus 120 mg of EPA or placebo for 180 days. Cognitive status was assessed using Mini-Mental State Examination (MMSE) and Abbreviated Mental Test (AMT) score.

**Results:**

MMSE and AMT scores were not different at the time of allocation [18.84 (5.37), 18.55 (5.12), (*P* = 0.70) and 4.81 (2.79) and 4.64 (2.77), (*P* = 0.67) respectively] and over 6 months between the ω-3 PUFA- and placebo- treated groups [18.57 (5.21), 18.39 (5.10), (*P* = 0.80) and 4.64 (2.77) and 4.48 (2.69) and (*P* = 0.67)]. The participants were categorized based on MMSE score into normal cognition, mild and moderate cognitive impairment. After multivariate adjustment, there was no significant difference among categorized groups regarding the ω-3 PUFA effect except in normal cognition group, that amount of decline in AMT in ω-3 poly unsaturated fatty acids (PUFAs) was less than placebo group.

**Conclusions:**

It seems that prescription of low dose ω-3 PUFAs for 6 months had no significant beneficial effects on improvement of cognition or prevention of cognitive decline in older people.

## Introduction

Cognitive impairment is a prevalent condition among the elderly and its burden tends to increase in parallel with increasing life expectancy, with an estimate of more than 81 million demented individuals by 2040 [1]. Hence the progressive and irreversible nature of the disease mandates identification of modifiable risk factors, which in turn facilitates primary prevention and early intervention.

The beneficial effects of ω-3 PUFAs, including eicosapentaenoic acid (EPA) and docosahexaenoic acid (DHA), have been demonstrated repeatedly in experimental studies [2-5]. Combined data from cross-sectional and longitudinal epidemiological studies point to the beneficial effect of ω-3 PUFA consumption on the primary prevention of age-related cognitive decline and dementia, notably Alzheimer’s disease (AD) [4-6]. However, there is a paucity of data from well-designed, randomized clinical trials concerning the beneficial effects of ω-3 PUFA supplementation on preventing dementia or slowing the progression of cognitive decline [4,7]. Freund-Levi et al. reported no beneficial effect of ω-3 fatty acid supplementation on cognitive function in patients with mild to moderate AD, except for a small subgroup of the study participants with the mildest forms of the disease [8]. Furthermore, DHA-enriched supplementation has been demonstrated to exert positive effects in patients with mild cognitive impairment (MCI), but not in those with established AD [9,10]. Although these results support the favorable effect of nutritional intervention in the earlier stages of the disease, either low- or high-dose ω-3 PUFA supplementation had no overall effect on cognitive performance amongst healthy individuals aged over 65 [11]. Similarly, the results of the largest randomized controlled trial conducted to date investigating the effect of ω-3 PUFA supplementation in cognitively healthy older people indicated no difference in cognitive function over a 24-month period [12].

Despite the multitude of epidemiological and preclinical studies concerning the neuroprotective properties of ω-3 PUFAs in the prevention and treatment of age-related cognitive decline and dementia, we decided to published this study for two reasons, first this study was conducted in about 5 years ago and at that time, there were very few clinical trials were carried out on effects of ω-3 PUFA on cognitive function in older people, the second reason, is that our study had a very good design and background variables were completely controlled because of its institutionalized based and the results of this study is useful in final conclusion, in systemic reviews which will conduct next, about ω-3 PUFA effectiveness on cognitive functions.

We chose normal cognitive elderly accompanied by mild to moderate cognitive impaired participants because we wanted to assess the preventive effect of ω-3 PUFAs on decrement of cognition in both normal cognitive and in mild and moderate cognitive impaired aged people. Moreover, we aimed to assess its efficiency in improvement of cognitive status in mild and moderate cognitive impairment. We chose low dose of DHA-EPA supplementation in order to evaluate the efficacy of dietary ω-3 PUFA doses on mental status. In other words the logic behind choosing such dose is its equivalence to normal diet of elderly Kahrizak Charity Foundation (KCF) residents. Especially the consumption of sea foods and other resources of ω-3 fatty acids such as nuts is very low in KCF residents (less than 3 times per year use of fish). On the other hand, based on our several years clinical experiments in KCF the higher dose of ω-3 PUFAs is difficult to tolerate by most of the elderly residents of this institute.

## Methods

### Participants

**Figure 1:**
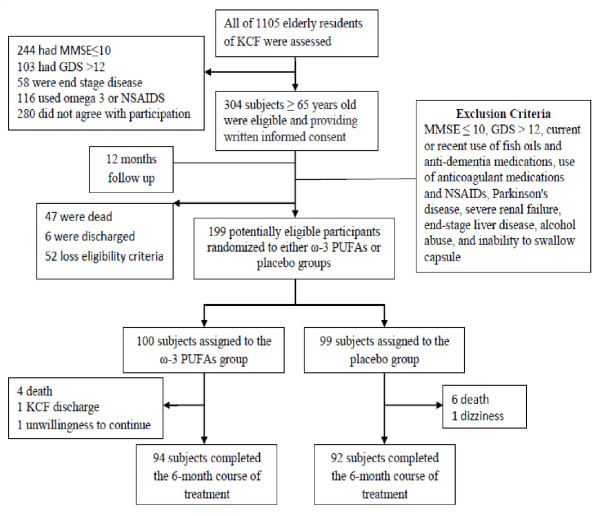
Flow diagram of progress through the study.

This study was conducted from September 2005 to March 2007. The study was designed as a single-center, randomized, double-blind, placebo-controlled trial. Participants were enrolled from KCF, a non-governmental charitable organization in which free-of-charge care is provided for elderly or physically handicapped individuals with no financial resources. Recruitment protocol is illustrated in Figure 1. Three hundred and four subjects out of 1105 KCF residents aged 65 years and over, who were able to understand and willing to provide written informed consent were enrolled. In order to obtain memory decline patterns [13] in normal cognitive and also various degrees of cognitive impaired aged people, the study participants were followed for up to 12 months while no intervention was implemented. At the end of this period, the participants were screened and those who did not meet the eligibility criteria were excluded. After that they were randomized into two groups as Placebo and PUFAs group. The exclusion criteria included severe dementia according to Diagnostic and Statistical Manual of Mental Disorders (DSM-IV) [14] and also Mini-Mental State Examination (MMSE) score equal or less than 10 [6,15] (if anyone met criteria of severe dementia in each of these scales, he/she was excluded), severe depression with geriatric depression scales (GDS) score equal or higher than 12 [8,16] current or recent (less than 8 weeks) use of fish oil supplements, current or recent (less than 12 weeks) use of anti-dementia medications, concomitant serious diseases, Parkinson’s disease, severe renal failure (serum creatinine > 2.5 mg/dl), end-stage liver disease, alcohol abuse, inability to swallow capsule, and use of anticoagulant medications.

### Allocation and intervention

One hundred ninety nine eligible participants were allocated in two groups. The treatment group received one capsule daily which contains 180 mg of DHA and 120 mg of EPA in 1 gram of cod liver oil, extracted from cold water fishes, plus glycerol and water. The placebo group received one capsule daily containing Medium Chain Triglycerides (MCTs), extracted from coconut oil, plus glycerol and water. Both fish oil and placebo capsules (manufactured by Zahravi Pharmaceutical Company) were completely the same in shape, color and packing. We used hard gelatin capsule to eliminate the odor and taste of fish oil in order to keep the study blinded. The participants consumed the capsules (placebo or ω-3 PUFAs) for 6 months (180 days).

### Primary outcome

The main outcome of this study was mental status which was assessed by two questionnaires. Mini mental state examination (MMSE) and Abbreviated Mental Test (AMT) were administered to assess cognitive status of participants, 12 months before initiation of clinical trial, another time just before allocation of participants into PUFAs and placebo groups, and at the end of the study. Score of the MMSE and AMT at time of the allocation was the basis of comparison between the PUFAs group and placebo group in this clinical trial. The MMSE is a 30-point questionnaire which consists of 11 questions assessing a broad range of cognitive functions including orientation, registration, attention and calculation, recall, and language [17]. It has been translated into Persian and validated for Iranian population [15].

The AMT was developed by Hodkinson in 1972 [18] (18). It has been widely used for clinical assessment of dementia in the elderly. It contains 10 questions with a maximum/optimal score of 10. The AMT includes tests of orientation in time and place, attention, and short as well as long term memory [18]. The validation of the AMT in Persian language in nursing homes has been approved in a study by the authors who are going to published [19]. Both tests were performed twice (in each time of evaluation) with about 7-day interval under standardized conditions and their average was used as final scores in data analysis in order to reduce the effect of potential confounders. The tests were implemented by a trained psychologist. The same person, who was blinded toward ω-3 PUFAs versus placebo treatment allocation, was responsible for repetition of the tests at the end of the study.

The participants were categorized into normal cognition, mild cognitive impairment, and moderate cognitive impairment based on MMSE scores assumed that MMSE > 21 as normal cognition, MMSE 17–21 as mild cognitive impairment and MMSE 11–16 as moderate cognitive impairment. The MMSE score equal and lower than 10 was assumed as severe cognitive impairment. (As we mentioned previously this group was excluded from the study at the time of allocation) [15]. It should mention that there were some participants with MMSE score > 10 at time of allocation whose scores dropped to less than 10 at the end of the study.

### Secondary outcomes

Plasma total cholesterol, low density lipoprotein cholesterol (LDL-C), high density lipoprotein cholesterol (HDL-C), triglyceride, high sensitivity C-reactive protein (CRP-hs) and fasting blood sugar (FBS) were measured twice just before allocation and at the end of the study. The changes in these measures were considered as secondary outcomes.

### Sample size calculation

The sample size required for this trial is 86 individuals per treatment group, with an assumption of 95% two-sided confidence interval, 80% power, and 10% difference in MMSE scores between ω-3 PUFAs- and placebo-treated groups being statistically significant (the logic of choosing of 10% effectiveness was that we think lower than it did not have clinical value). Allowing for 10% loss during over 6 months of intervention, the total sample size required for the study is 190 individuals calculated with Open Epi-2. In the present study, 199 participants, who gave full, informed written consent, were enrolled.

### Data collection

At baseline demographic characteristics including age and gender were recorded. In addition past medical history of diseases such as previous medical diagnoses including diabetes mellitus, dyslipidemia, hypertension, coronary artery disease and stroke was recorded. Histories of medications in use, smoking and socioeconomic status, such as educational level were also obtained from medical records and also directly through the participant interview. All of the above measurements and medical histories were repeated just before the allocation. Blood pressure was measured twice just before the allocation a trained nurse with automatic sphygmomanometer Omron 7 according to The Seventh report of the Joint National Committee on Prevention, Detection, Evaluation, and Treatment of High Blood Pressure (JNC7) [20]. Anthropometric measurements were performed using standard protocols and techniques. Weight and height were measured in light indoor clothing without shoes. Body mass index (BMI) was calculated as weight in kilograms divided by height in square meters. Following overnight fasting, blood samples were collected prior to instantiation and after the period of intervention. Blood samples were centrifuged at 2000 rpm for 10 minutes at room temperature. Plasma aliquots were divided into micro tubes and stored at -32°C until analyses. Subsequently total cholesterol, HDL-C, LDL-C, triglyceride, and FBS levels were measured by enzymatic method (Pars Azmun, Iran) using auto-analyzer (Hitachi 902, Osaka, Japan). CRP-he was measured using an enzyme linked immunosorbent assay (ELISA) method (Cayman USA).

### Diet, medications and life styles

To assess dietary habits, just before allocation and at the completion of the trial, two trained nutritionists completed the Food Frequency Questionnaire on three separate days, including two weekdays and one weekend. All items provided on food record were estimated by household units and converted to standard servings using Iranian household units [21]. Energy and nutrient intake were computed by Nutritionist III software (version 3.5.2, N-squared Computing, Salem, Ore). During the study period, the diets of the participants remained unchanged. Moreover, the participants were advised to continue their medications, except for non-steroidal anti-inflammatory drugs (low dose aspirin was an exception), with no change in their physical activity and lifestyle habits, including smoking.

### Randomization and blindness protocols

The participants were allocated into two groups receiving either ω-3 PUFAs or placebo. Randomization was performed according to “Random Number Generation Method” (100 subjects in the ω-3 PUFAs group and 99 subjects in the placebo group). The randomization process was performed by one trained staff who did not participate in any other processes, such as providing fish oil or placebo capsules, evaluation of the participants, data entry or analyses. Both the study participants and other trial staff were completely blinded to treatment assignment.

Fish oil and placebo capsules were exactly similar in shape and taste. They were packed in similar single package and uncovered at the time of consumption. The medications were provided by one person with no medical education based on the A or B code on the package. To assess compliance and ensure complete adherence to the study protocol, the participants were instructed to consume the daily delivered capsules under direct supervision. If any subject did not use the prescribed drug any day, it was returned to project management office to record the amount and the reason not to consume the drug.

### Statistical analyses

The Kolmogorov-Smirnov test was used to assess the normal distribution of continuous variables. At baseline, categorical and nonparametric variables were compared using nonparametric χ^2^ and Mann–Whitney U tests and parametric variables were compared using t-test. Intra-rater analysis was performed using Pearson’s correlation between repeated MMSE and AMT scores at baseline, just before the allocation, and at the end of the study period. The primary and secondary outcomes were analyzed by comparing the change in scores between the two groups using t-test and univariate analysis with adjustment for *education level*, *history of diabetes mellitus*, *history of cardiovascular disease*, *body mass index*, *total cholesterol and triglyceride* which differed between the two groups at the time of allocation (Table 1). Repeated measures analysis was used to test differences in scores during 12 months follow up as such, as the time of allocation and after the 6 months intervention. Statistical significance was assessed using χ^2^ and Fisher’s exact test. A *P* value less than 0.05 was considered to be statistically significant. The scores of different components of the MMSE were compared between the two groups using t-test and univariate analysis with adjustment for above mentioned confounders.

**Table 1:** Characteristics of the participants at time of allocation

		**ω-3 PUFAs**	**Placebo**	***P*-value**
		**(n=100)**	**(n=99)**	
**Age (years)**		74.13 ± 9.96*	75.17 ± 8.70*	0.43
**Male (%)**		44	46.5	0.72
**Education (%)**	**Illiterate**	67	69.7	0.179
	**Primary**	16	17.2	
	**Secondary**	9	12.1	
	**Higher**	8	1	
**Smoking (%)**	**Never smoker**	74	72.7	0.78
	**Ex-smoker**	8	12.1	
	**Current smoker**	18	15.2	
**BMI (kg/m**^**2**^**)**		25.70 ± 5.50*	25.24 ± 5.10*	0.53
**Diabetes mellitus (%)**		9	17.2	0.09
**Dyslipidemia (%)**		7	7.1	0.31
**Hypertension (%)**		51	52.5	0.70
**History of CVD (%)**		41	31.3	0.15
**History of stroke (%)**		17	13.1	0.45
**GDS-15**		5.39 ± 3.45*	5.72 ± 3.27*	0.48
**CRP-hs (mg/L)**		4.67 ± 5.12*	3.37 ± 3.97*	0.04†
**Total cholesterol (mg/dl)**		197.64 ± 43.56*	184.79 ± 39.38*	0.03†
**LDL-C (mg/dl)**		116.84 ± 29.77*	110.66 ± 27.13*	0.12
**HDL-C (mg/dl)**		44.28 ± 14.13*	43.96 ± 11.84*	0.86
**Triglyceride (mg/dl)**		159.46 ± 85.05*	137.06 ± 63.37*	0.03†
**FBS (mg/dl)**		100.13 ± 30.62*	99.47 ± 35.85*	0.89

*PUFA*, polyunsaturated fatty acid; *BMI*, Body mass index; *CVD*, Cardiovascular disease; *GDS*, Geriatric depression scale; *CRP-hs*, High sensitivity C-reactive protein; *LDL-C*, Low density lipoprotein cholesterol; *HDL-C*, High density lipoprotein cholesterol; *FBS*, Fasting blood sugar.

*Values are expressed as mean ± SD.

†*P* < 0.05 is considered statistically significant.

### Ethical considerations

The study was conducted in accordance with good clinical practice and ethical principles of the Declaration of Helsinki. Written informed consents were signed by eligible participants and their legally authorized representatives (in cases who had an MMSE score lower than 22) [15] after reading it completely or reading it, by their trusted individual and also verbal explanation about the aims and the outcome as such, as randomization, probability of allocation into a placebo group and probable side effect by a trained nurse prior to enter to study. The participants had the right to leave the study any time they desired without having any responsibility. Also the subjects were followed up for any harm effect. For the participant who received the criteria of dementia if had indications for anti-dementia treatment after finishing the study this drug were prescribed for them. The protocol of this study was approved by the ethics committee of Endocrinology and Metabolism Research Institute (EMRI) and KCF ethics committees.

## Results

One hundred ninety nine subjects (109 women and 90 men) were enrolled in the present study. We found a declining trend in the MMSE and AMT scores during 12 months flowed up pre-allocation of both the normal cognitive and demented participants. But this decrement in AMT scores was not significant during this period. MMSE scores decreased from 19.19 ± 5.48 to 18.69 ± 5.23 (P < 0.01) and ATM scores from 4.84 ± 3.02 to 4.72 ± 2.77 (*P* = 0.41). On the other words we detected a 0.5 score decrement in MMSE score and a 0.12 score AMT score per year in population of our study before intervention.

The characteristics of our study participants at time of randomized allocation demonstrated in Table 1. One hundred eighty six of the 199 participants (93.4%) completed the clinical trial study. The reasons for premature withdrawal were death (n = 10), subject release (n = 1), the primary complaint of dizziness (n = 1), and unwillingness to continue to cooperation (n = 1). The number of withdrawals was comparable between two groups (*P* = 0.55). The results of dietary assessment just before the allocation and at the time of finishing the trial are demonstrated in Table 2. There were no differences in dietary intakes before and after intervention and between the ω-3 PUFAs- and placebo-treated groups (Table 2).

**Table 2:** **The mean and standard deviation of the dietary intakes in ω**-**3 PUFAs**- **and placebo**-**treated groups before and after intervention**

	**ω-3 PUFAs**		***P*** **value**	**Placebo**		***P*** **value**
	**Before**	**After**		**Before**	**After**	
**Energy (kcal/d)**	1709.85 ± 302.29	1711.10 ± 283.19	NS	1719.46 ± 274.11	1727.28 ± 222.91	NS
**Carbohydrate (%)**	52.04 ± 2.47	52.74 ± 3.83	NS	51.86 ± 3.71	52.69 ± 5.17	NS
**Protein (%)**	13.25 ± 1.13	13.11 ± 2.35	NS	13.04 ± 0.67	12.84 ± 1.41	NS
**Fat(%)**	34.69 ± 2.57	34.14 ± 4.05	NS	35.09 ± 3.63	34.46 ± 5.10	NS
**SFA (g/d)**	19.65 ± 4.09	19.17 ± 4.92	NS	20.23 ± 3.15	19.85 ± 3.68	NS
**MUFA (g/d)**	26.76 ± 5.82	25.69 ± 6.36	NS	27.90 ± 5.11	28.52 ± 3.96	NS
**PUFA (g/d)**	13.18 ± 3.28	13.01 ± 3.24	NS	13.99 ± 2.64	13.80 ± 3.01	NS
**Linoleic Acid (g/d)**	12.30 ± 3.12	12.27 ± 3.23	NS	13.01 ± 2.55	13.90 ± 3.12	NS
**α**-**Linolenic Acid (g/d)**	0.28 ± 0.05	0.28 ± 0.13	NS	0.28 ± 0.05	0.27 ± 0.08	NS

*SFA*, Saturated fatty acid; *MUFA*, monounsaturated fatty acid; *PUFA*, polyunsaturated fatty acid; *NS*, not significant.

The reported side effects were of mild, including gastrointestinal discomforts (flatulence), i.e. mild diarrhea in the ω-3 PUFAs (n = 3) and placebo (n = 1) groups.

### Primary outcome

The MMSE scores just before allocation and at the end of the trial are summarized in Table 3. Each of the results which was reported herein are the average of two MMSE score assessment which was performed with a 7 day interval just before allocation and also another 2 other time after the 6 months intervention. Intra-rater correlation coefficients between two time measurements of MMSE scores at baseline (12 months before allocation) were 0.91. As such, as Intra-rater correlation coefficients between two time assessments of MMSE just before allocation was 0.93, and at the end of the study this correlation was 0.90. Regarding the AMTS scores, the results were 0.98, 0.95, and 0.98, respectively. In the beginning of the trial, the mean MMSE scores were 18.84, [standard deviation (SD) = 5.37] and 18.55, SD = 5.12 (*P* = 0.70) and the mean AMT scores were 4.81, SD = 2.79 and 4.64, SD = 2.78 (*P* = 0.68) in the ω-3 PUFAs- and placebo-treated groups, respectively.

**Table 3:** Effects of ω3 PUFAs on different levels of cognitive impairment

	**Categorization of cognitive status based on MMSE scores**	**At the time of allocation**			**Final (6 months after intervention)**			**P value**	**P value**	**Chang scores in ω3 PUFAs group**	**Chang scores in placebo group**	**P value of change scores**
		**ω3-PUFAsn=100**	**Placebon=99**	**P value**	**Pairedt-test**	**Placebon=92**	**P value**	**Pairedt-test**	**Pairedt-test**			
		**Mean (SD)**	**Mean (SD)**	**T-test**	**Mean (SD)**	**Mean (SD)**	**T-test**	**ω3**	**Placebo**	**Mean (SD)**	**Mean (SD)**	
**MMSE**	**Normal**	26.07 (2.02)	25.21 (2.45)	0.16	25.75 (2.05)	24.69 (2.63)	0.11	<0.01	<0.01	-0.36 (0.61)	-0.50 (0.64)	0.43
		N=29	N=28		N=26	N=27						
	**Mild**	17.95 (1.64)	18.07 (1.71)	0.74	17.81 (1.71)	17.83 (1.97)	0.98	0.01	0.11	-0.19 (0.44)	-0.22 (0.86)	0.81
		N=41	N=42		N=40	N=40						
	**Moderate**	13.06 (1.72)	12.82 (1.75)	0.53	13.00 (1.68)	12.48 (1.85)	029	0.04	0.03	-0.14 (0.35)	-0.28 (0.61)	0.32
		N=30	N=29		N=28	N=25						
	**Total participants**	18.84 (5.37)	18.55 (5.12)	0.70	18.57 (5.21)	18.39 (5.10)	0.80	<0.01	<0.01	-0.22 (0.48)	-0.32 (0.74)	0.29
		N=100	N=99		N=94	N=92						
**AMT**	**Normal**	8.79 (1.05)	8.35 (1.76)	0.27	8.61 (2.00)	7.44 (1.76)	0.03	0.64	<0.01	-0.15 (1.66)	-0.89 (1.25)	0.07
		N=29	N=28		N=26	N=27						
	**Mild**	3.87 (1.00)	3.81 (1.47)	0.80	3.82 (1.31)	4.02 (2.18)	0.62	0.65	0.63	-0.10 (1.39)	0.15 (1.96)	0.51
		N=41	N=42		N=40	N=40						
	**Moderate**	2.23 (0.43)	2.27 (0.45)	0.71	2.14 (0.35)	2.04 (0.20)	0.20	0.26	0.03	-0.11 (0.50)	-0.24 (0.52)	0.35
		N=30	N=29		N=28	N=25						
	**Total participants**	4.81 (2.79)	4.64 (2.78)	0.68	4.65 (2.90)	4.49 (2.70)	0.70	0.38	0.11	-0.12(1..28)	-0.26 (1.54)	0.49
		N=100	N=99		N=94	N=92						

PUFAs: Poly unsaturated fatty acids, SD: Standard deviation, MMSE: mini mental examination state, AMT: abbreviated mental test, ANOVA: analysis of variance.

Likewise, following the completion of the trial, the mean MMSE scores were 18.57 SD = 5.21 and 18.39 SD = 5.10 (*P* = 0.80) and the mean AMT scores were 4.65 SD = 2.90 and 4.49 SD = 2.70 (P = 0.70), respectively. During the 6-month trial, the mean MMSE scores decreased significantly in both the ω-3 PUFAs- and placebo-treated groups (*P* < 0.01); whereas the decrease in the mean AMT scores was neither statistically significant in the ω-3 PUFAs- nor in the placebo-treated groups (*P* = 0.38 and *P* = 0.11, respectively). The mean change of MMSE scores by did not differ significantly between the ω-3 PUFAs- and placebo-treated groups, i.e. -0.22 SD = 0.48 *vs*. -0.32 SD = 0.74 (*P* = 0.29), respectively. After adjustment for age, sex, smoking, cholesterol and CRP serum levels no change in significance and mean levels (*P* = 0.30).

Similarly, the mean change of AMT scores was not statistically different between the groups, i.e. -0.12 SD = 1.28 *vs*. -0.26 SD = 1.54 (*P* = 0.49) in the ω-3 PUFAs and placebo groups, respectively.

The participants were categorized at time allocation based on MMSE scores into 3 categories (normal cognition, mild cognitive and moderate cognitive impaired) and the effect of low dose ω-3 PUFAs was compared with placebo in these 3 groups. We could not detect any difference in MMSE score between ω-3 PUFAs and placebo group. We only could detect near significant less decrement in AMT scores among normal cognitive participants in ω-3 PUFAs group than placebo group (Table 3).

No significant difference was detected between the changes in the scores of the components of the MMSE in ω-3 PUFAs group in comparison to placebo, before and after adjustment for confounding factors (data not shown).

Subgroup analysis was performed for females and males. The results demonstrated that there was no gender difference regarding the effect of ω-3 PUFAs on cognitive status was detected (*P* = 0.42 and *P* = 0.50 in males and females respectively). The effect of ω-3 PUFAs and placebo were assessed on MMSE and AMT sub-items. It was not detected any significant change in scores about sub- items of both AMT and MMSE.

The risk of development of cognitive impairment based on MMSE scores through 6 months follow up was assessed among normal cognitive subjects (29 subjects in ω-3 PUFAs and 28 subjects in placebo groups). Nobody in the ω-3 PUFAs group and one of the participants in placebo group were developed cognitive impairment (relative risk = 0.49; CI 95% = 0.37-0.66) but this not clinically significant result.

### Secondary outcomes

We measured plasma cholesterol, triglyceride, HDL-C, LDL-C, CRP-hs and FBS levels just prior to and just after the trial. All of these variables decreased in the ω-3 PUFAs group except for CRP-hs which increased (2.19 ± 7.78). In the placebo group, LDL-C, HDL-C and Cholesterol decreased and CRP-hs, FBS and Triglyceride increased. The decreasing of triglyceride levels in ω-3 group in comparison to the placebo group was significant (with a mean change -3.38 ± 65.79 in the PUFAs group and 21.82 ± 70.62 in the placebo group; *P* = 0.01) (Table 4).

**Table 4:** **Secondary outcomes of the controlled clinical trial of low**-**dose ω**-**3 PUFA supplementation and placebo** (**by comparison of mean and standard deviation of changes in two groups**)

	**Change in ω-3 PUFAs group**	**Change in placebo group**	***P-value***
**Total Cholesterol (mg/dL)**	-8.03 ± 35.06	-2.20 ± 40.01	0.29
**LDL-C (mg/dL)**	-1.44 ± 10.30	-2.51 ± 7.70	0.43
**HDL-C (mg/dL)**	-1.41 ± 27.21	-1.56 ± 25.29	0.96
**Triglyceride (mg/dL)**	-3.38 ± 65.79	21.82 ± 70.62	0.01Ψ
**hsCRP (mg/L)**	2.19 ± 7.78	2.61 ± 6.03	0.69
**FBS (mg/dL)**	-2.70 ± 33.30	1.27 ± 27.46	0.38

*UFA*, polyunsaturated fatty acid; *LDL-C*, Low density lipoprotein cholesterol; *HDL-C*, High density lipoprotein cholesterol; *CRP- hs*, High sensitivity C-reactive protein; *FBS*, fasting blood sugar. Ψ with Mann–Whitney test.

### Loss to follow-up analysis

Thirteen subjects (6.5%) were lost to the follow-up (6 subjects in from the ω-3 PUFAs group and 7 subjects from the placebo group). There was no difference in observed characteristics of those who were lost to follow up and the remainder of participants.

Sensitivity analysis of missing data was performed based on AMT cut point. It is based on 4 assumptions: all of the failed subjects, improved in AMTS categorized group all of them exacerbated in AMTS categorized group, the subjects who were lost from the ω-3 PUFAs group improved and those of the placebo group exacerbated, and vice versa. No significant difference was observed except for the third assumption. Since the occurrence of third assumption is too weak, it could be neglected (Table 5).

**Table 5:** Sensitivity analysis of the missing data assumptions

	**ω-3 PUFAs group**		**Placebo group**		
	**No.**	**%**	**No.**	**%**	***P-*** **value**
**All failed participants were improved**					
***Improved***	9	9	8	8.1	0.78^*^
***No change***	87	87	85	85.8	
***Exacerbated***	4	4	6	6.1	
**All failed participants were exacerbated**					
***Improved***	3	3	1	1	0.48^§^
***No change***	87	87	85	85.8	
***Exacerbated***	10	10	13	13.2	
**The failed Participants from ω**-**3 PUFAs group were improved and those from placebo group were exacerbated**					
***Improved***	9	9	1	1	0.00^§^
***No change***	87	87	85	85.8	
***Exacerbated***	4	4	13	13.2	
**The failed Participants from ω**-**3 PUFAs group were exacerbated and those from placebo group were improved**					
***Improved***	3	3	8	6.1	0.19^*^
***No change***	87	87	85	85.8	
***Exacerbated***	10	10	6	6.1	

*PUFA*, polyunsaturated fatty acid.

*χ^2^ test.

§Fisher’s exact test.

## Discussion

We observed that, low dose ω-3 PUFA supplementation had no overall therapeutic effect on mental status among the elderly with mildly to moderately impaired cognition. We found that declines of AMT scores may was lesser in ω-3 PUFAs than the placebo group among the normal cognition participants; Although it is seems that it not has any clinical significance because not approved by the MMSE scores and the difference between two groups is too small that not had any importance. Furthermore, it was not proved to be an effective short term preventive measure against cognitive decline among the elderly with normal cognition.

We also found that the PUFAs have no improving effect on any components of MMSE. In other words, the short term, low dose of PUFAs have not any effect on time and place orientation, data registry, recall, attention, naming, repetition, writing, reading ability an execution function. Another finding of our study as a secondary outcome was that the triglyceride level changes in placebo and PUFAs group was different and the median of triglyceride levels in the PUFA group did not change while it increased in the placebo group.

The OmegAD study, as our study found that in patients with mild to moderate AD ω-3 PUFAs had no beneficial effects on cognitive performance, even after daily supplementation of 1700 mg of DHA and 600 mg of EPA for 6 months. However, in patients with very mild AD (MMSE score >27), statistically significant treatment effects were observed in the MMSE score, MMSE sub-items “Delayed word recall” and “Attention”, and ADAS-COG sub-item “Delayed word recall” [8]. Notwithstanding, the possibility of chance findings cannot be excluded. Similarly, patients with mildly to moderately depressed mood showed an overall negligible benefit from daily supplementation of 850 mg of DHA and 630 mg of EPA for 12 weeks [22]. In another study which was published recently even high doses of EPA- and DHA- rich ω-3 fatty acid supplementation had no significant effect on cognitive and mood function of healthy young adults [23].

On the contrary, the results of a pilot study of daily supplementation of 240 mg arachidonic acid (ARA) and DHA for 90 days revealed a significant improvement of immediate and delayed memories in subjects with mild cognitive impairment [10] (9). Based on experimental models of age-dependent cognitive dysfunction, both ω-3 and ω-6 PUFAs play indisputable roles in neuronal growth and development of neural network connectivity. Therefore, combined supplementation with ARA and DHA could not differentiate the beneficial effects of either supplement on cognitive dysfunction.

Despite the lack of an overall *therapeutic* benefit of ω-3 PUFA supplementation on cognitive performance, the rationale for fish oil supplementation as a *preventive* measure has recently been evaluated in healthy elderly subjects (MMSE score > 21) [11]. Compared with placebo, either low dose (400 mg/d) or high dose (1800 mg/d) of DHAEPA supplementation did not hinder age-related cognitive decline in a 26 week period.

However, post hoc analyses revealed significant improvement in the cognitive domain of “Attention” mainly among APOE-4 carriers and male subjects. Similarly, no potential beneficial effect of daily 200 mg EPA plus 500 mg DHA for 24 months has been reported in cognitively healthy adults [12]. However, the lack of decline in cognitive function in the placebo arm may have contributed to an underestimation of the potential advantages of ω-3 PUFAs supplementation in cognitively healthy older people [12]. In summary, it seems that these discrepancies among the findings of several studies may be somewhat due to difference between prescription dose, different levels of consumption of PUFAs at baseline, diversity of Pharmacogenomics of population (especially difference in the prevalence of APOE-4 allele in different populations), discrepancy between the ratio of EPA to DHA that was prescribed in different studies, diversity in duration of prescription of PUFAs in these studies and also in methods of evaluation of cognitive function and the age of the participants.

The triglyceride lowering effect of PUFAs which was observed in our study has been approved in many studies [24,25].

We assessed the cognitive status with MMSE and AMT. Although MMSE may not seem very suitable for assessment of cognitive status in illiterate older people [26] (26), we used an age-education specific norm [27] of MMSE which would resolve such problem. We used a Persian validated version of the MMSE for categorization of cognitive status. We also used AMT for a more comprehensive assessment of cognitive status. An unpublished study in the Kahrizak Charity Foundation has shown that the AMT is a good instrument for assessment of cognitive status in illiterate older people [19]. Elderly individuals represent the largest population at risk for nutritional deficiencies due to aging-associated alteration in nutritional requirements, impaired digestion and absorption of nutrients, and altered dietary habits accompanied by social, behavioral and lifestyle modifications [28]. The American Heart Association has recommended two oily fish servings (≈8 ounces) per week for overall health and cardiovascular care. The combined DHA and EPA equivalence of such fish consumption is approximately 250–300 mg/day [29]. Furthermore, the authors of the prospective Framingham Heart Study gave an estimated daily intake of 180 mg DHA in the protected group with the upper quartile blood DHA levels [30]. There are very few available data on the dietary intake of the elderly [31,32] and similar studies, particularly the current levels of fish consumption, among the Iranian elderly population are lacking. In the present study, the results of the food frequency questionnaire and the monthly 3-course menu provided at the institution indicate the nutritional gap between actual and targeted intakes (equal or less than 2 times per year). We examined the effect of 300 mg of combined DHA and EPA, which corresponds to the recommended dietary ω-3 PUFAs intakes. The rationale for choosing the low dose of DHA-EPA supplementation was to evaluate the efficacy of recommended dietary ω-3 PUFA intakes in either preventing or slowing the progression of age-related cognitive decline prior to the administration of high “pharmacological” doses. However, our study was designed when no clinical trial on the effect of ω-3 PUFAs on cognitive performance had been published. Therefore, compared to similar studies, the chosen dose was apparently low and dose-ranging clinical trials are recommended to close the controversy. Although several studies indicated that a higher proportion of EPA to DHA is beneficial for psychiatric abnormalities and depression, another study in the elderly has shown that the similar proportions of DHA to EPA to our study could be effective in the treatment of mild to moderate depression [33].

The results of this study do not provide definitive evidence in support of the beneficial effects of low dose ω-3 PUFA supplementation on cognitive performance among the elderly with normal or mildly to moderately impaired cognition. Given that the slowly emerging beneficial effects of specific nutrients mandate decades of exposure, one limitation of the current study was the absence of a group with consumption of higher doses of ω-3 PUFAs. Therefore, we were unable to detect effects of high dose ω-3 in comparison to low dose. Moreover, Serum fatty acid measurement was not feasible in our setting. Such measures could help us a better judgment of compliance to ω-3 PUFAs consumption. Another limitation may be regarded to a ratio of EPA and DHA, which was prescribed in this study. Because a study has reported that DHA-rich PUFAs improve prefrontal oxygenation by increasing of the concentration of Oxy-hemoglobin and total level of hemoglobin compared to placebo; although no effect on cognitive tasks was detected [34].

In another study it has been found that the 6 months administration of low dose PUFAs with doses similar to our study may increase the hemoglobin level in older people. Although in that study Oxy-hemoglobin levels were not measured, but their finding about the level of hemoglobin is similar to that of Jackson and et al. [35]. Thus, as it is true in other randomized clinical trials, the lack of beneficial effects may not suffice to draw further conclusions. Therefore, we recommend further trials to be conducted with an extended period of intervention and follow-up starting at midlife.

## Competing interests

We declare that there is no conflict of interest with any financial organization regarding the material discussed in the manuscript. This study was supported by the Endocrinology and Metabolism Research Institute of Tehran University of Medical Sciences grant numbers MP10/0125, 2005. The ω-3 and Placebo Capsules were supplied free of charge by Zahravi Pharmaceutical Company. This company did not have any role in designing, data gathering, analysising and writing of this manuscript.

## Authors’ contributions

MJM: He participated in designing the study and writing the draft of the manuscript. MH: She has major participation in writing the draft of the manuscript. FS: He designed the study and he was the main analyzer of the data and writing the draft of the manuscript. MM: She has contributed in designing and carrying out the study. NN: She has contributed in designing and was the responsible for the data collection. NM: She has contributed in data collection and writing the draft of the manuscript. MG: She has contributed in data collection and writing the draft of the manuscript. YT: He has contributed in data collection of the study. ZB: She has contributed in data collection of the study. BL: He has contributed in designing and approved the final revision of the manuscript. SA: She has contributed in data analyzing of study and writing the draft of the manuscript. MA: She has contributed in analyzing and writing the draft of the manuscript. SMA: He has contributed in designing and writing the draft of the manuscript. BN: She has contributed in data gathering and writing the draft of the manuscript. HF: He has been the principle investigator of the study and had a major role in designing and conducting the study and writing the manuscript. He approved the manuscript finally too. All authors read and approved the final manuscript.
